# Reservoir Regulation for Ecological Protection and Remediation: A Case Study of the Irtysh River Basin, China

**DOI:** 10.3390/ijerph191811582

**Published:** 2022-09-14

**Authors:** Dan Wang, Shuanghu Zhang, Guoli Wang, Yin Liu, Hao Wang, Jingjing Gu

**Affiliations:** 1 School of Hydraulic Engineering, Dalian University of Technology, Dalian 116024, China; 2 State Key Laboratory of Simulations and Regulations of Water Cycles in River Basins (SKL-WAC), China Institute of Water Resources and Hydropower Research (IWHR), Beijing 100038, China; 3 School of Environment and Ecology, Jiangsu Open University, Nanjing 210036, China

**Keywords:** ecological protection, ecological regulation, hydrological process, hydrodynamic model, valley forests and grasslands

## Abstract

Hydrological processes play a key role in ecosystem stability in arid regions. The operation of water conservancy projects leads to changes in the natural hydrological processes, thereby damaging the ecosystem balance. Ecological regulation is an effective non-engineering measure to relieve the influence of water conservancy projects on ecosystems. However, there are still some problems, such as an insufficient understanding of hydraulic processes and difficulty evaluating the application effects. In this study, the theory of ecological reservoir regulation coupled with hydrological and ecological processes was examined and ecological protection and remediation were investigated using the valley forests and grasslands in the Irtysh River Basin as a case study. The results demonstrated that (1) to meet the demand of the hydrological processes in the valley forests and grasslands, in terms of ecological regulation, the peak flow and flood peak duration of the reservoir, named 635, in the Irtysh River Basin should be 1000 m^3^ s^−1^ and 168 h, respectively, and the total water volume of ecological regulation should be 605 million m³. Ecological regulation can guarantee that the floodplain range reaches 64.3% of the core area of ecological regulation and the inundation duration in most areas is between 4–8 d; (2) an insufficient ecological water supply would seriously affect the inundation effects. The inundation areas were reduced by 2.8, 5.1, 10.3, and 19.3%, respectively, under the four insufficient ecological water supply conditions (528, 482, 398, and 301 million m^3^), and the inundation duration showed a general decreasing trend; (3) the construction of ecological sluices and the optimization of the reservoir regulation rules could effectively relieve the influences of an insufficient ecological water supply. At water supply volumes of 528 and 482 million m^3^, the regulation rules should assign priority to the flood peak flow; at water supply volumes of 398 and 301 million m^3^, the regulation rules should assign priority to the flood peak duration. Consequently, this study provides a reference for ecological protection in arid regions and the optimization of ecological regulation theories.

## 1. Introduction

Water is the most important environmental factor that determines the structural integrity and functional stability of inland river basin ecosystems in arid regions [1,2,3]. As the dominant factor in ecological processes, hydrological processes support complex ecosystem functions and services [4] and serve as the main driver of productivity and sustainable development in terrestrial ecosystems [5,6]. Valley forests and grasslands are the main components of inland river basin ecosystems in arid regions, and their reproduction and renewal, community succession, and spatial distribution patterns are closely related to river hydrological processes [2,7]. In turn, river hydrological processes play a critical role in maintaining the stability of ecosystems [8,9,10] by replenishing the groundwater to meet the ecological water demand of valley forests and grasslands in arid regions [11,12]. With the increasing water demands of humans, the construction of water conservancy projects has become a key measure for solving the water demand problem [13,14,15,16,17]. Nevertheless, the operation of water conservancy projects has changed the natural hydrological processes [18,19,20], and it is typically difficult to balance the protection requirements of ecosystems and water environments [21,22], resulting in adverse changes in the materials, energy, and biology of water bodies [23,24] and affecting natural river ecosystems [25,26,27,28]. Especially in arid regions, the competition between economic and ecological water utilization is escalating [29], which worsens the problem of ecological protection and remediation.

The negative impacts of water conservancy projects on ecosystems can be avoided, mitigated, or compensated to some extent by engineering, biological, and management measures [30]. Reservoir regulation can compensate for or mitigate the resulting ecological issues, and it is the most important non-engineering measure for the ecological remediation of large rivers at home and abroad [31,32,33,34]. Due to the differences and priorities of ecological protection objectives, studies on ecological regulation have not yet agreed upon a uniform method, and attention has mainly been paid to considering ecological flows in reservoir regulation, reducing the negative impacts of conventional reservoir regulation on the environment, and maintaining and improving the ecosystem [35,36,37]. To maintain the health of ecosystems, specific and relatively stable ecological processes are required. Currently, in studies on ecological regulation regarding ecological protection and remediation, problems such as an inadequate understanding of the hydrological process requirements at key ecological stages and difficulty in assessing ecological regulation performance still occur [38]. These phenomena will lead to ecological regulations that cannot reasonably meet the demand, with unreasonable water resource allocation and other problems. For valley forests and grasslands in arid regions, natural flooding on the floodplain during the flood season is an essential hydrological process that is needed to meet the water demand for reproduction and growth. However, the construction and operation of water conservancy projects have weakened the flow in the river channels during the flood season, which has reduced the chance of flooding in the floodplain and decreased the inundation range and duration, resulting in a water shortage and disruption in the reproductivity of valley forests and grasslands. Consequently, the balance of the ecosystem is destroyed. Therefore, the core protection and remediation strategy for valley forests and grasslands is to shape the flood peak process through reservoir regulation to promote flooding on the floodplain during the flood season and expand the inundation range, thereby restoring this essential hydrological process to meet the water demand of valley forests and grasslands at key ecological stages.

The Irtysh River originates in the arid region of northwestern China, and its special topography and climate have created a unique valley forest and grassland ecosystem. With the construction and operation of water conservancy projects, the hydrological processes in the basin of the Irtysh River have changed drastically, and the water demand for the reproductivity and growth of valley forests and grasslands cannot be satisfied, which has caused many ecological issues. A hydrodynamic model is a powerful tool for studying the responses of river hydrological processes to reservoir regulation. However, in the Irtysh River Basin, the complex underlying surface conditions, including the complex topography of the river valleys, huge replenishment from the surface water to the groundwater, and the flooding of the floodplain, result in frequent alternations in the climate in the floodplain between wet and dry within short periods. This greatly increases the difficulty of flood simulation and analysis. To date, a hydrodynamic model of the study area has not been studied. Therefore, in this paper, a two-dimensional (2D) hydrodynamic model was constructed to simulate the flooding process in the basin of the Irtysh River. The difficulty in simulating the flooding process lies in the huge number of grids and nodes at a large scale and the inability to reasonably calculate the flooding process with frequent alternations between the wet and dry boundaries and the interaction between the surface water and groundwater. TELEMAC is a finite element model with unstructured meshes for simulating surface water that was developed by Laboratoire National d’Hydraulique et Environnement at the Research Department of Electricité de France, and it has good application for 2D hydrodynamic simulation [39,40]. Its parallel computation can also significantly improve a model’s computational efficiency [40]. In this study, the ecological protection and remediation of valley forests and grasslands in the basin of the Irtysh River was the study objective. We aimed to construct a 2D hydrodynamic model using the 2D module of TELEMAC to propose an ecological regulation method to meet the needs of the hydrological processes of the valley forests and grasslands, to evaluate the effect of the ecological protection and remediation, and to propose measures to ameliorate the problem of insufficient ecological water supply. We set out to (1) determine the key parameters of the reservoir that were needed to meet the requirements for flooding in the key ecological section and improve water utilization efficiency; (2) quantify the effects of ecological regulation on the floodplain, vegetation coverage, and grass yield; and (3) analyze the impacts of insufficient ecological water supply on the ecological regulations and propose solutions. The results of this study can provide a scientific basis for realizing ecological protection and remediation in arid regions and improving the theoretical system of ecological regulation.

## 2. Materials and Methods

### 2.1. Study Area

**Figure 1:**
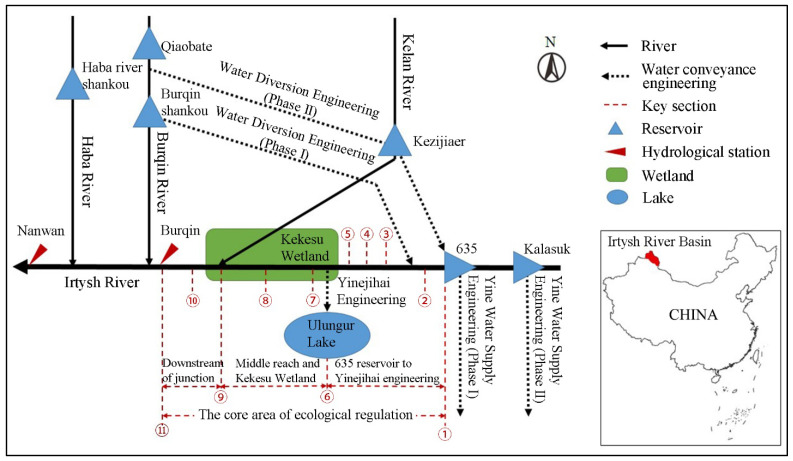
The topography of the water conservancy projects in the basin of the Irtysh River, China (red number: the number of the key section).

As the sixth longest river in the world, the Irtysh River originates from the southern slope of the Altai Mountains in northwestern China, flows through Kazakhstan and Russia, and joins the Ob River, eventually flowing into the Arctic Ocean. In China, the Irtysh River basin lies between 47–48° N, 85°30’–90°28’ E and has a 633 km main stream and a basin area of about 52,500 km^2^. Affected by the topography, the basin has a unidirectional plume-shaped asymmetric water system, with all the tributaries (including the Kelan River, Burqin River, and Haba River, as shown in Figure 1) flowing from north to south into the main stream. The basin is in an inland arid region, with low precipitation, strong evaporation, and an unbalanced hydrology. The middle and lower courses of the Irtysh River, especially the middle course, have wide valleys, meandering streams, wide and shallow riverbeds, terraces on both banks, and flat terrain. There is no embankment built on either side of the river, and natural floods form floodplains every year, which has nurtured a unique terrestrial ecosystem of valley forests and grasslands. The vegetation types from the rivers to the high terraces occur in the order of swampy wetland, lowland meadow, riparian forest, woodland, shrub, high terrace scattered woodland, and sparse shrub. The valley forests and grasslands cover a total area of 1401 km^2^, including 378 km^2^ of forests and 1023 km^2^ of grasslands.

To meet the human water demand, several reservoirs and water diversion and transfer projects have been built in the basin to achieve water allocation. In addition, a water diversion project, Yinejihai Engineering, was built to meet the water needs of Ulungur Lake, outside the basin. The construction and operation of water conservancy projects have caused significant changes in the hydrological processes in the middle course of the Irtysh River, resulting in the reduction of floodplain probability, area, and duration. The changes in the hydrological processes have affected the balance of the valley forest and grassland ecosystem, such as obstructing the flooding of forest species and leaving insufficient water supply for forests and grasslands.

The ecological regulation of reservoirs and the remediation of the essential hydrological processes of the valley forests and grasslands are of great significance for the protection and remediation of the ecosystem. The core area of ecological regulation is from the 635 Reservoir, located in the Irtysh River, and Kezijiaer Reservoir, located in the Kelan River, to the Burqin section in the middle course of the basin. The channel lengths of the Irtysh River and Kelan River in the core area are 221 and 112 km, respectively, and the total area of the valley forests and grasslands is 1071 km^2^. The key sections of the study area are shown in Figure 1 and Table 1. The main aims of the ecological regulation are to regulate the hydrological processes in the middle course through the 635 Reservoir, promote the flooding of the floodplain, expand the inundation range, and satisfy the water demand for the reproductivity and growth of the valley forests and grasslands.

### 2.2. Data Sources

**Figure 2:**
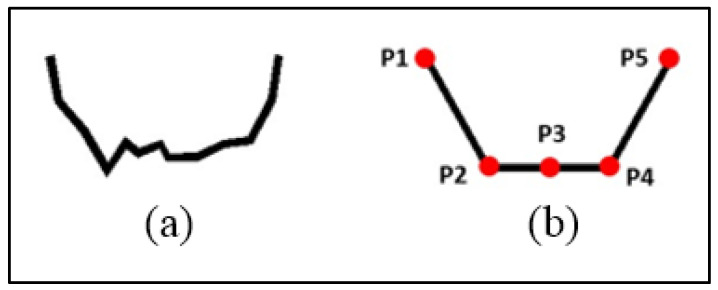
The channel section shape: (**a**) measured section; (**b**) generalized section. Red dot: key point in the section.

This study was conducted based on a 2D hydrodynamic model. To construct this model, geological, hydrological, meteorological, and soil infiltration data were required. The model was first calibrated using the observation data from 2016, and then the model simulation accuracy was validated using the observation data from 2017 and the remote sensing image data (2016–2017) from the SPOT-6 satellite, which was developed by Astrium GEO-Information Services in France and launched in 2012, and the Environment-1 satellite, which was developed by China Aerospace Science and Technology Corporation and launched in 2008. The data and precision are shown in Table 2. The geological data were obtained from the geological survey results. Then, a trapezoidal section was used to generalize the channel section to ensure the quality of the channel mesh division and the computational efficiency of the model, as shown in Figure 2. The labels P1 and P5 in Figure 2 indicate the riverbanks, P3 indicates the middle thread of the section, and P2 and P4 indicate the midpoint between the riverbanks and the middle thread of the section. For the main channel, the model used a spacing of 10–15 m to discretize the channel according to the channel width and a spacing of 7–10 m to discretize the irrigation channel. Based on the measured section data, interpolation was performed on the river and irrigation channels to obtain the elevations at P1–P5. Additionally, the hydrological data included the outflow processes of the 635 and Kezijiaer reservoirs at a 2 h interval, and interpolation was performed on the daily water levels and flows of the Burqin Hydrological Station and the daily flows of Yinejihai Engineering to obtain the water level and flow processes with a 2 h interval. Furthermore, the evaporation data were obtained from the daily evaporation observation data of the meteorological station, and the daily average evaporation of the study area was calculated using the large water surface evaporation data, which was obtained from Altai Meteorological Services. In terms of the infiltration data, we embedded three soil moisture monitoring stations using LD-TS600 (Laiende Intelligent Technology Company Limited, China) in the core area for hourly monitoring, and the daily average infiltration in the study area was calculated. In TELEMAC, the evaporation and infiltration were merged with the water loss and were only calculated in the area that was covered by water.

### 2.3. Methods

#### 2.3.1. Theory of Reservoir Regulation

**Figure 3:**
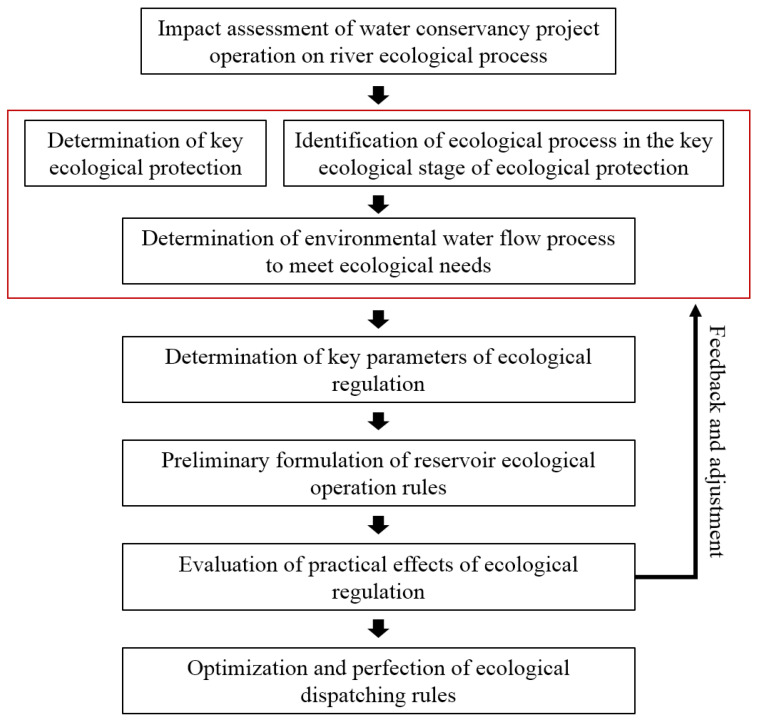
The research process of reservoir regulation.

There is a coupling relationship between the hydrological and ecological processes that influences and regulates them. As one of the operation objectives, reservoir regulation aims to incorporate the protection and restoration of the ecosystem into the daily operation of the reservoir and to shape the key hydrological processes, which is conducive to achieving the important ecological protection objectives. The research process of reservoir regulation is shown in Figure 3. The key point is to establish a coupling relationship between the ecological and hydrological processes and to determine the key parameters. For the valley forests and grasslands in the Irtysh River Basin, the core aim of the ecological regulation is to shape the flood peak process to promote flooding on the floodplain and to use the least amount of water to achieve optimal flooding effects. The key parameters of ecological regulation include the flood peak flow and water volume. Therefore, a two-dimensional hydrodynamic model was used as a tool to determine the reservoir regulation parameters.

#### 2.3.2. Computation Principle and Reliability Evaluation of the Two-Dimensional Hydrodynamic Model

The TELEMAC-2D model [41] was used to construct the 2D hydrodynamic model. In this model, the Saint Venant equation is used to describe the motion of changing and non-constant water flows in the watercourses and other shallow bodies of water with free surfaces, and it consists of a continuity equation that reflects the law of conservation of mass and a momentum equation that reflects the law of conservation of momentum. The model computation is performed using a stepwise algorithm based on the characteristic curve method. The computation process can be divided into two parts: one for solving the horizontal advection terms and the other for solving the propagation, diffusion, and source terms. Through computation, the average water depth and flow rate in the vertical direction were obtained, and the flooding process was simulated in a 2D space.

The error and Nash–Sutcliffe efficiency factor (*NSE*) [42] are widely used to quantify the reliability of a hydrodynamic model of flow processes and peak simulation. In this study, the flood peak flow error and *NSE* were used to evaluate the model simulation accuracy. The flood peak flow error was set as the absolute value of the error between the simulated and measured flows, and when the error was lower than 20%, the model was considered to meet the accuracy requirement. *NSE* was calculated using Equation (1), and the model met the accuracy requirement when *NSE* was greater than 0.8. The equation for the *NSE* coefficient is as follows:

(1) $$NSE=1-\frac{\sum_{i=1}^{n}{\left(X_{obs,i}-X_{\mathrm{mod}el}\right)}^{2}}{\sum_{i=1}^{n}{\left(X_{obs,i}-\bar{X_{obs}}\right)}^{2}}$$

where $X_{obs}$ is the measured value, $X_{\mathrm{mod}el}$ is the simulated value, $\bar{X_{obs}}$ is the average measured value, and *n* is the length of the data series.

**Figure 4:**
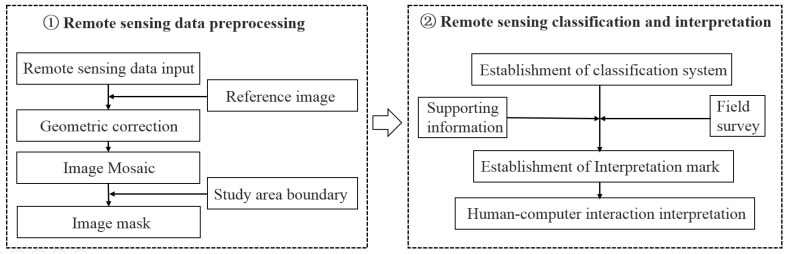
The technical paths for extracting the floodplain range that are based on remote sensing images.

Additionally, the advancements in remote sensing and geographic information system technologies provide very convenient data support for water surface area extraction [43,44,45]. Compared with the interpretation of conventional water surfaces, the interpretation of the valley floodplain area varies due to the vegetation growth and high vegetation coverage on the water surface. Therefore, in this study, unsupervised classification based on the ISODATA algorithm was used to interpret the remote sensing images, and the calculation steps are shown in Figure 4. In addition, the reliability of the hydrodynamic model was evaluated based on the floodplain range.

#### 2.3.3. Key Parameters for the Hydrological Process Remediation

Regulating the outflow of the 635 Reservoir to achieve the essential hydrological processes is the main measure that could be implemented to meet the water demand of the valley forests and grasslands at key ecological stages. Reservoir regulation can promote the flood plain and expand the inundation range, and the key parameters include the flood peak flow and water volume. For the flood peak flow, a 2D hydrodynamic model was used to describe the water levels at the critical ecological section of the middle course of the Irtysh River under different flood peak outflows from the 635 Reservoir. The suitable flood peak flow of the 635 Reservoir should meet the floodplain in most sections. For the water volume, both ecological regulation effects and water utilization efficiency need to be considered, and these can be determined through the variations in the inundated area under the flood peak flow of the 635 Reservoir. When the inundated area increases rapidly, it indicates that the duration of flood peak can be increased to improve the ecological regulation effects. When the growth rate of the submerged area decreases, it indicates that the ecological regulation effects of the same amount of water decrease, and the 635 Reservoir should stop discharging.

## 3. Results

### 3.1. Calibration and Validation of the Two-Dimensional Hydrodynamic Model

#### 3.1.1. Model Establishment

**Figure 5:**
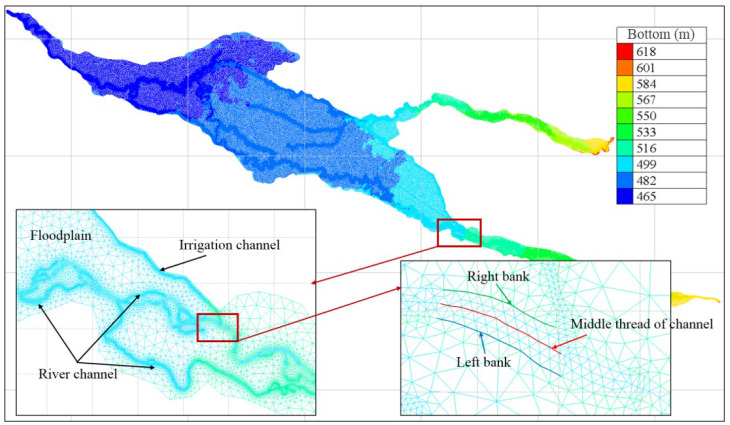
A schematic diagram of the mesh division of the two-dimensional hydrodynamic model.

The TELEMAC-2D model sets the model input files and numerical parameters using keywords and stores them in *.cas format as a steering file. In this study, a 2D hydrodynamic model was built based on the core area of ecological regulation, with a total area of 1039.0 km^2^. Considering the computational efficiency of the model and the influence of microtopography, the model used a mesh with an accuracy of 300 m and encrypted the river channel, irrigation channel, and floodplain near the channels. Then, meshes with an accuracy of 60, 1, and 7 m were used for the floodplain near the channels, main river channel, and irrigation channel and part of the main river channel with a relatively narrow width, respectively. In the model, the number of nodes was about 290,000, and the number of mesh cells was about 580,000. The mesh division results are shown in Figure 5. The computational duration of the model was 10 d, and multiple trial computation results showed that a step size of 5 s could ensure both the computational stability and efficiency of the model.

#### 3.1.2. Model Calibration

**Figure 6:**
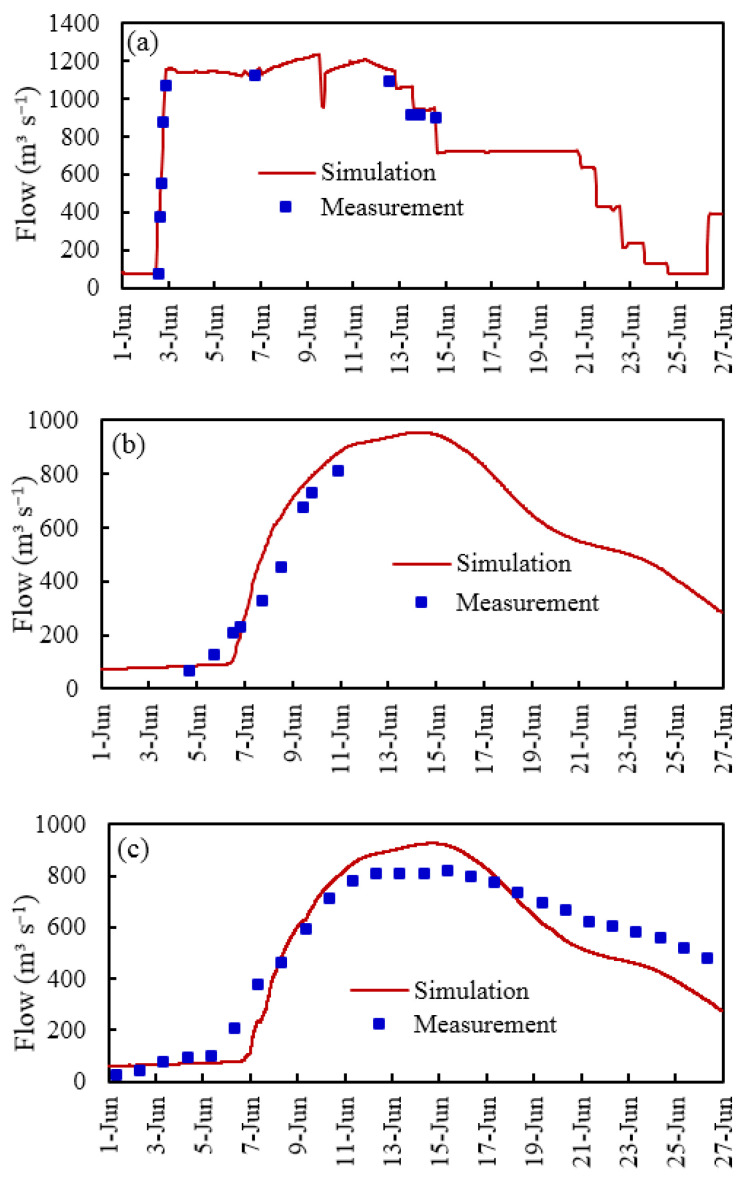
A comparison between the simulated and measured data for the model calibration: (**a**) Key section ②; (**b**) Key section ⑩; (**c**) Key section ⑪; data from 2016.

The reference sections for model calibration were the section of the Burqin Hydrological Station (Key section ⑪) at the outlet boundary, the Sarbulak section (Key section ②) downstream of the inlet boundary, and the section of the Halagou Mudao Bridge (Key section ⑩) downstream of the junction of the Irtysh River and Kelan River. The comparison between the model calibration results of the sections and the measured data is shown in Table 3 and Figure 6. The flood peak flow simulation errors at the three monitoring sections of the Burqin Hydrological Station, Sarbulak, and Halagou Mudao Bridge were 11.7, 2.1, and 8.3%, respectively, and *NSE* was 0.88, 0.85, and 0.85, respectively. All the above values were within the allowable range of accuracy, indicating that the model calibration results were reasonable and could effectively simulate the flooding process.

#### 3.1.3. Model Validation

**Figure 7:**
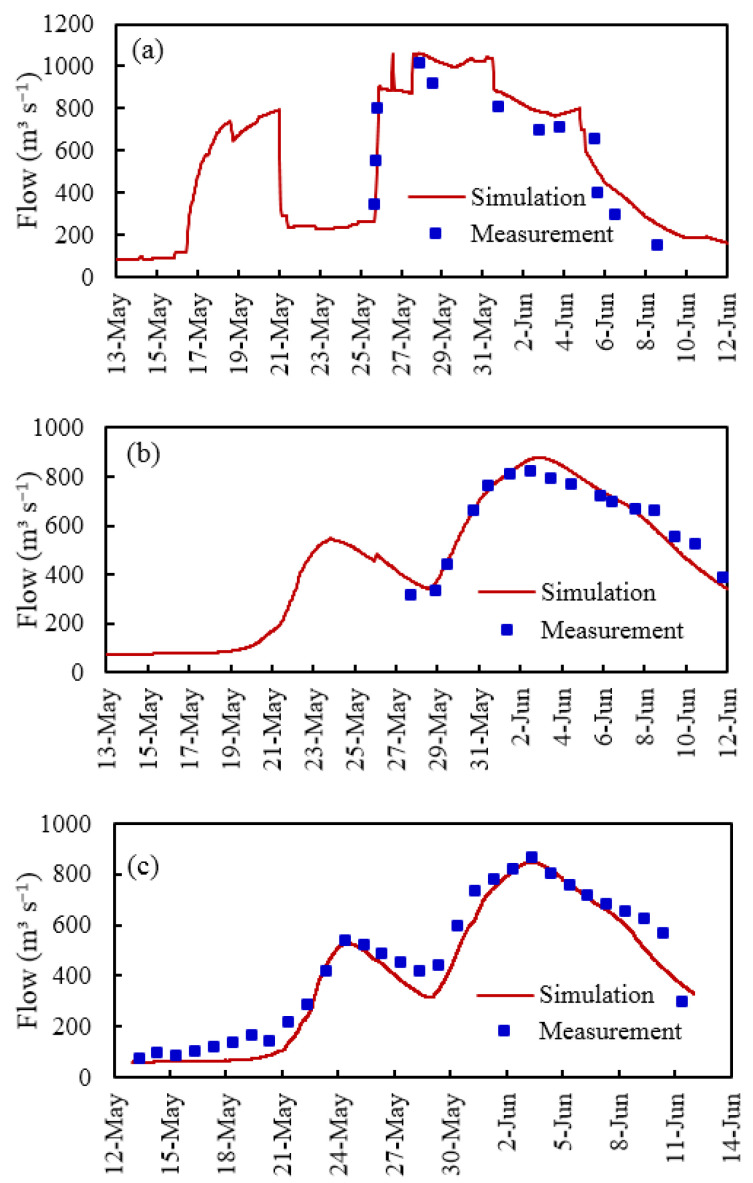
A comparison between the simulated and measured data for the model validation: (**a**) Key section ②; (**b**) Key section ⑩; (**c**) Key section ⑪; data from 2017.

**Figure 8:**
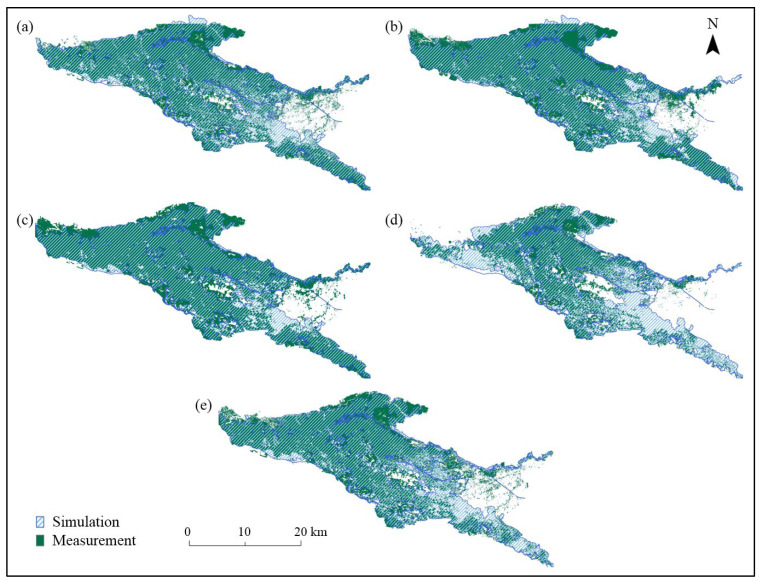
The measured and simulated inundation ranges in the core area: (**a**) 9 June 2016; (**b**) 12 June 2016; (**c**) 14 June 2016; (**d**) 22 May 2017; (**e**) 3 June 2017.

Among the measured hydrological data in 2017, only the flooding process during the second regulation was measured for the Sarbulak and Halagou Mudao Bridge sections. The comparison between the model simulation results and the measured data for each reference section is shown in Table 4 and Figure 7. For the flow process of the Burqin Hydrological Station section, *NSE* was 0.94, and the simulation errors of the first and second flood peak flows were −2.4% and −2.5%, respectively. For the Sarbulak and Halagou Mudao Bridge sections, *NSE* was 0.85 and 0.93, respectively, and the simulation errors of the second flood peak flow were 4.2 and 6.0%, respectively. Based on the available remote sensing image data, this study interpreted the flooding situation on 9 June, 12 June, and 14 June in 2016 and 22 May and 3 June in 2017, and the results were compared as shown in Table 5 and Figure 8. The simulation error of the inundation area was observed to be within the allowed range, and the simulation results of the inundation range were consistent with the results that were interpreted from the remote sensing images, indicating that the model met the accuracy requirements and was both reasonable and reliable.

### 3.2. Key Parameters for the Hydrological Process Remediation

#### 3.2.1. The Flood Peak Flow of Ecological Regulation

Table 6 describes the water levels at the critical section of the middle course of the Irtysh River under different flood peak outflows from the 635 Reservoir. The results show that most sections of the Irtysh River were flooded when the flood peak outflow of the 635 Reservoir reached 1000 m^3^ s^−1^. Additionally, according to the historical experience of ecological regulation, if the flood peak outflow of the 635 Reservoir is higher than 1200 m^3^ s^−1^, the farmland in some areas will be inundated. Therefore, the appropriate flood peak flow for ecological regulation with the 635 Reservoir is 1000 m^3^ s^−1^.

#### 3.2.2. The Water Volume of Ecological Regulation

**Figure 9:**
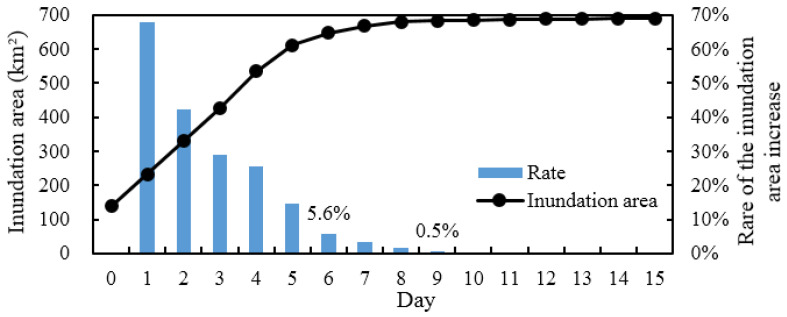
The inundation area versus the flood peak duration.

Figure 9 shows the changes in the inundation area in the study area under the continuous flood peak outflow of 1000 m^3^ s^−1^ from the 635 Reservoir. The results show that the growth rate of the inundation area was predicted to be high in the early stage of ecological regulation and then gradually decrease as the flood peak flow duration increased. In the first 5 d, the growth rate of the inundation area was more than 10%. On the 6th day, the inundation area increased to 646.6 km^2^, and the growth rate decreased to 5.7%. On the 8th day, the inundation area increased to 679.9 km^2^, while the growth rate decreased by less than 1%. To ensure the ecological regulation effects and improve the water utilization efficiency, the flood peak duration from the 635 Reservoir should be between 6 and 8 d. Therefore, the reasonable flood peak duration from the 635 Reservoir is 7 d (168 h) on average, and the ecological regulation water volume is 605 million m^3^.

### 3.3. Evaluation of the Ecological Regulation Effects

#### 3.3.1. Inundation Area and Range

**Figure 10:**
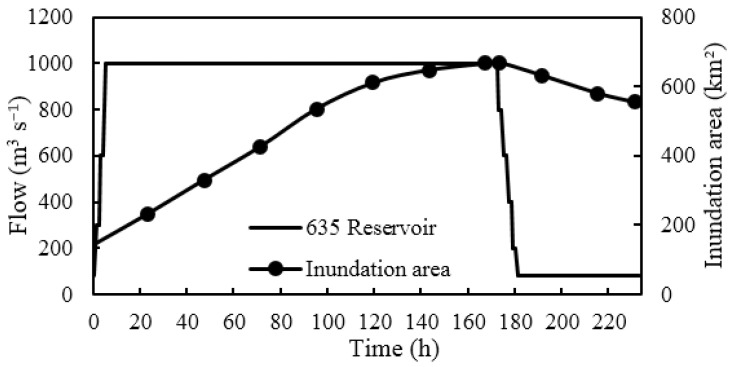
Variation in the inundation area during the computational period.

**Figure 11:**
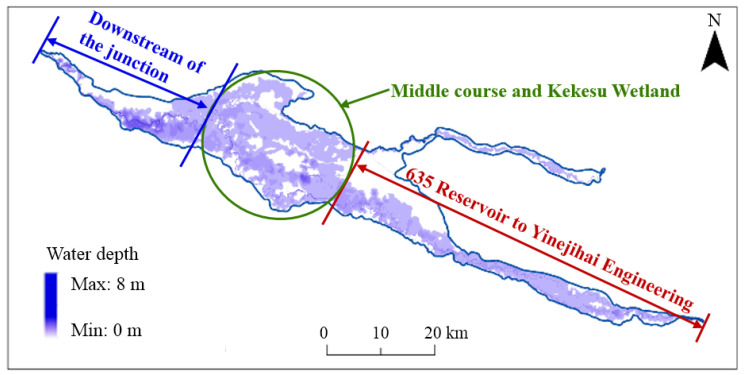
The maximum flooding range when the ecological water supply volume is 605 million m^3^.

The changes in the inundation area during the calculation period are shown in Figure 10. The inundation area kept increasing at a fast rate in the first 5 d after the start of ecological regulation and decreased gradually from the 5th day, reaching the maximum inundation area at the 174th hour, with a lag of 2 h after the end of ecological regulation. After the end of regulation, the inundation area gradually decreased. With an ecological water supply of 605 million m^3^, the maximum inundation area was 668.5 km^2^, and the inundation range reached 64.3%, as shown in Figure 11. The inundation area from the 635 Reservoir to the inlet of Yinejihai Engineering was 222.8 km^2^, with a percentage of 54.3%. The inundation range on the two banks of the bottomland in the middle course and the Kekesu Wetland was 327.1 km^2^, with a percentage of 72.4%. Additionally, the inundation area downstream of the junction of the Irtysh River and the Kelan River was 118.6 km^2^, with a percentage of 67.0%.

#### 3.3.2. Distribution of the Inundation Duration

**Figure 12:**
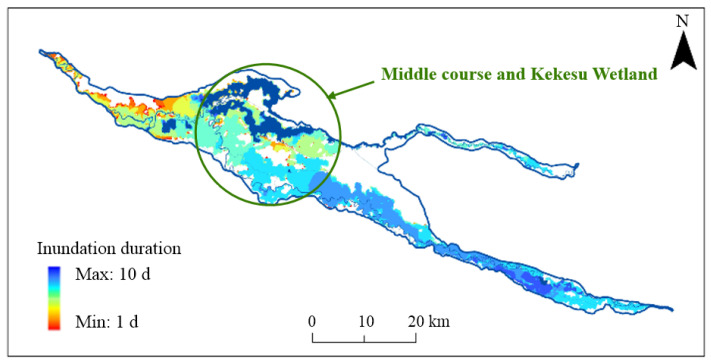
The distribution of the inundation duration during the computational period when the ecological water supply volume is 605 million m^3^.

**Figure 13:**
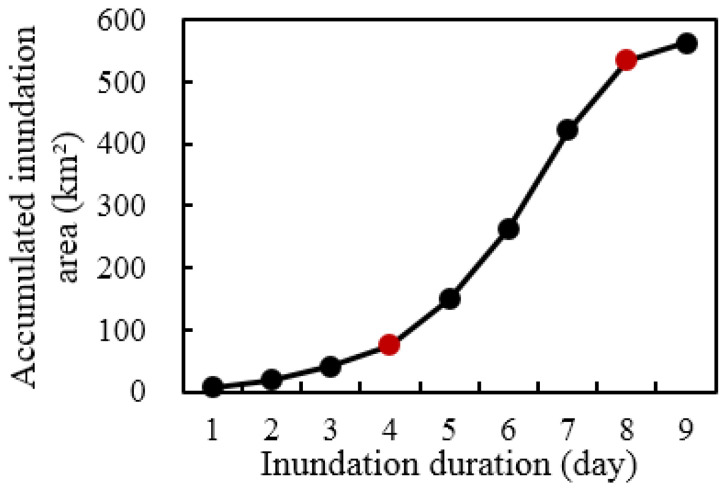
The accumulated inundation area of the inundation duration during the computational period (red dot: turning point of the curve).

The distribution of the inundation duration during the calculation period is shown in Table 7 and Figure 12. An inundation duration of 10 d indicates the areas that were always flooded, such as the rivers and ponds, and an inundation duration of 0 d indicates the areas that did not flood during the ecological regulation period. Figure 13 shows the curve of the accumulated inundation area with an inundation duration of 1–9 d. The inflection points at 4 d and 8 d indicate that most of the areas were flooded for 4–8 d, with a total inundation area of 562.9 km^2^. This was mostly distributed in the core area of the middle stream and the Kekesu Wetland. The area that was inundated for 7 d was the largest part of the study area, with an area of 159.9 km^2^, and an area of 301.0 km^2^ was inundated for 7 d or more.

#### 3.3.3. Variation in the Vegetation Coverage

Based on the research results, ecological regulation practices were carried out under the direction of the Construction and Management Authority of the Irtysh River from 2016 to 2019. The regulations regulated the hydrological processes of the river by regulating the discharge from the 635 Reservoir, to promote flooding on the floodplain and meet the water demand for the growth of the valley forests and grasslands.

**Figure 14:**
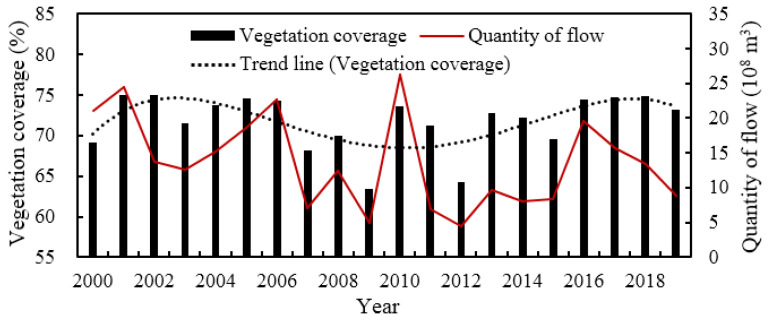
The annual variation in the vegetation coverage.

**Figure 15:**
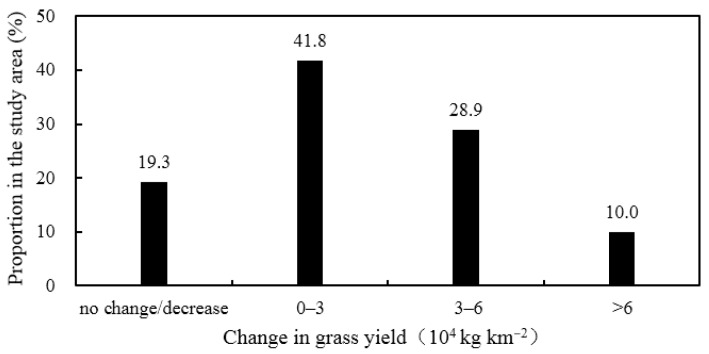
The statistics of the spatial variation in grass yield after the implementation of ecological regulation.

Vegetation coverage and grass yield are effective indicators of the health of valley forest and grassland ecosystems [46]. Figure 14 shows the variation in the vegetation coverage in the study area and the discharge during the flood period for 635 Reservoir from 2000 to 2019, and there was a good correlation between them, with a correlation coefficient of 0.62. The vegetation coverage showed an overall decreasing trend from 2000 to the implementation of ecological regulation, based on the trend line with a fourth-order regression. From 2007 to 2015, the vegetation coverage was generally low, with an average of 69.5%, which was 3.8% lower than that from 2000 to 2006, and the vegetation coverage fluctuated greatly, with minimums of 63.4% in 2009 and 64.2% in 2012. After the implementation of ecological regulation, the average vegetation coverage recovered to 74.3% in 2016–2019, which exceeded the average in 2000–2006. However, during this period, the average discharge from the 635 Reservoir during the flood period was less than that from 2000 to 2006. This indicated that the parameters of ecological regulation in this study were reasonable and could effectively restore the ecological environment by saving water resources. Figure 15 shows the statistics of the spatial variation in grass yield after the implementation of ecological regulation. Compared with the pre-regulation state, more than 80% of the study area showed an increase in grass yield, among which 41.8% of the area had an increase of 0–3 × 10^4^ kg km^−2^, 28.9% had an increase of 3 × 10^4^–6 × 10^4^ kg km^−2^, and 10.0% had an increase of more than 6 × 10^4^ kg km^−2^. The results demonstrated that the vegetation coverage and grass yield of the valley forests and grasslands were restored to some extent and that the ecosystem remediation effect was good.

### 3.4. Impacts of Insufficient Ecological Water Supply on Ecological Regulation

To alleviate the water shortage in the economic zone on the northern slope of the Tianshan Mountains in Xinjiang Uygur Autonomous Region, a water conveyance project, Yine Water Supply Engineering, was constructed to divert the water from the Irtysh River to meet the urban and industrial water needs of the Urumqi Economic and Technological Development Zone and the oil fields in northern Xinjiang Uygur Autonomous Region, and the project is expected to be completed by 2030. The operation of the Yine Water Supply Engineering project will result in competition between the economic and ecological water supply objectives. Therefore, according to the project design, this study discussed the possible impacts of insufficient ecological water supply on ecological regulation under four working conditions (528, 482, 398, and 301 million m^3^).

#### 3.4.1. Impacts on the Inundation Area and Range

**Figure 16:**
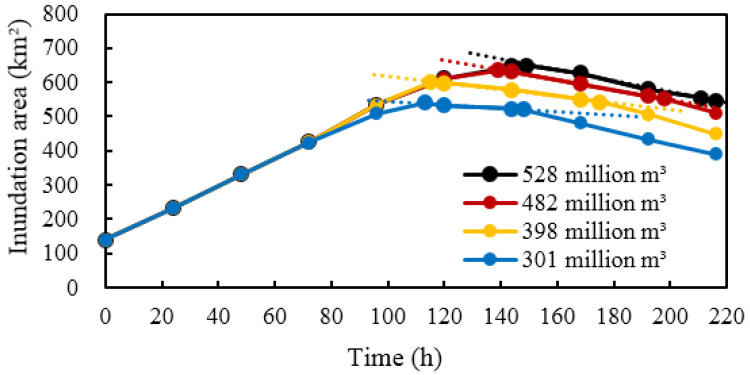
The variation in the inundation area under different ecological water supply volumes.

A shortage in the ecological water supply would lead to a corresponding decrease in the flood peak duration of the 635 Reservoir. Figure 16 and Table 8 show the variation in the inundation area and the comparison between the maximum inundation areas under different ecological water supply conditions. Compared with the conditions when the target of 605 million m^3^ ecological water supply was satisfied, the maximum inundation areas decreased by 18.6, 33.8, 69.0, and 128.8 km^2^ when the water supply volumes were reduced to 528, 482, 398, and 301 million m^3^, respectively. Additionally, the maximum inundation area was reduced by nearly 20% when the water supply volume was reduced to 301 million m^3^. As can be observed from the variation in the inundation area, when the water supply volume was greater than 398 million m^3^, the maximum inundation area occurred at the end of the flood peak duration from the 635 Reservoir. When the water supply volume was less than 398 million m^3^, the flood peak duration from the 635 Reservoir decreased, but it had little impact on the maximum inundation area. Furthermore, in the water-receding stage, the lower the water supply volume, the smoother the decrease in the inundation area in the early stage.

**Figure 17:**
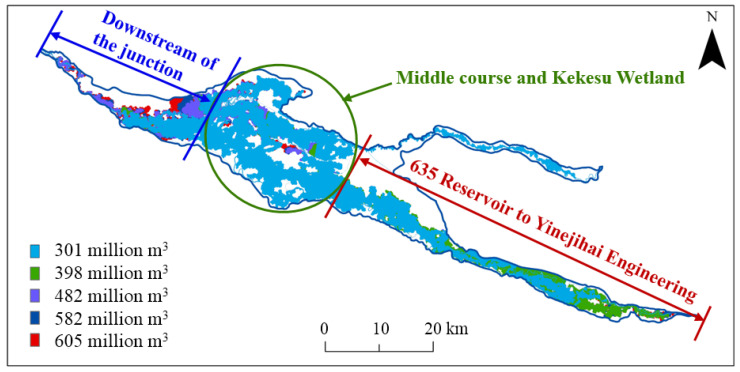
A comparison between the maximum inundation ranges under different ecological water supply volumes.

Figure 17 compares the maximum inundation range under different ecological water supply volumes, and the areas that were more affected by insufficient ecological water supply were the river valley on both banks from the 635 Reservoir to Yinejihai Engineering and downstream of the junction of the Irtysh River and Kelan River. For the area from the 635 Reservoir to Yinejihai Engineering, the range that was impacted was relatively small when the water supply volume was more than 398 million m^3^, but when the water supply volume decreased to 301 million m^3^, the inundation area in this area changed significantly. This was mainly because the flood discharge flow of the 635 Reservoir had already been reduced and the flood upstream of the Irtysh River had gradually returned to the channel. Downstream of the junction of the Irtysh River and the Kelan River, the range of the areas that were not inundated increased significantly as the water supply decreased, but the change in the inundation range was stable when the water supply volume was less than 398 million m^3^.

#### 3.4.2. Impacts on the Inundation Duration and Distribution

Table 9 depicts the distribution of the inundation duration under different ecological water supply volumes during the calculation period. When the ecological water supply target of 605 million m^3^ was met, the floodplain with an inundation duration of 7 d covered the largest area, and the range of the floodplain with an inundation duration of more than 7 d was 301.0 km^2^. When the water supply volumes were reduced to 528, 482, 398, and 301 million m^3^, the inundation duration of the floodplain was reduced, and the ranges of the floodplain with an inundation duration of more than 7 d decreased to 238.6, 199.9, 155.7, and 121.5 km^2^, respectively, reductionss of 20.7, 33. 6, 48.3, and 59.6%, respectively.

**Figure 18:**
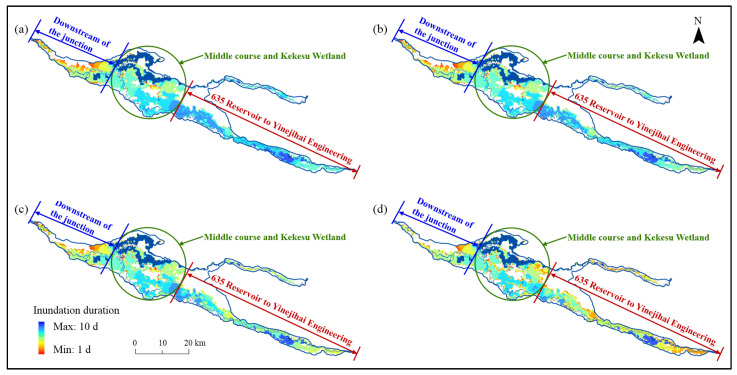
The distribution of the inundation duration under different ecological water supply volumes: (**a**–**d**) indicate 528, 482, 398 and 301 million m^3^, respectively.

The distribution of the inundation duration under different ecological water supply volumes is shown in Figure 18. According to the variation characteristics, the distribution of the inundation duration was divided into three parts for the discussion: the part from the 635 Reservoir to Yinejihai Engineering (part 1), the middle course and Kekesu Wetland (part 2), and downstream of the junction of the two rivers (part 3).

When the water supply volume decreased from 528 million m^3^ to 482 million m^3^, the change was mainly reflected in the shortening of the inundation duration for the bottomland on both sides of the river channel of part 1. For parts 1 and 2, the change in the inundation duration distribution was small except in the areas that could not be flooded.

When the water supply volume decreased to 398 million m^3^, the inundation duration changed significantly for the bottomland on both sides of the river channel of part 1. Furthermore, except for the bottomland that was close to the river channel, where inundation could last for more than 6 d, the inundation duration of the more distant bottomland was shortened to less than 5 d. Moreover, the inundation range of part 2 showed a small change that was mainly reflected in the shortening of the inundation duration at the edge of the floodplain. The distribution of the inundation duration of part 3 mainly showed a decrease in the inundation range and a consequent decrease in the inundation duration.

When the water supply volume decreased to 301 million m^3^, the inundation duration was significantly shortened, except for the Kekesu Wetland, where the inundation could last for more than 6 d in some areas. In addition, most of the bottomland areas on both sides of the river channel of part 1 were only inundated for less than 4 d. The inundation duration at the edge of the inundated area of part 2 was significantly shortened, and the inundation duration of most of the areas upstream of the wetland was reduced from 5 to <4 d. Moreover, the inundation range of part 3 was reduced, and the areas that were previously inundated for <4 d were almost free of flooding.

### 3.5. Ecological Regulation Optimization Measures Based on the Ecological Sluices

#### 3.5.1. Geographic Locations of the Ecological Sluices

**Figure 19:**
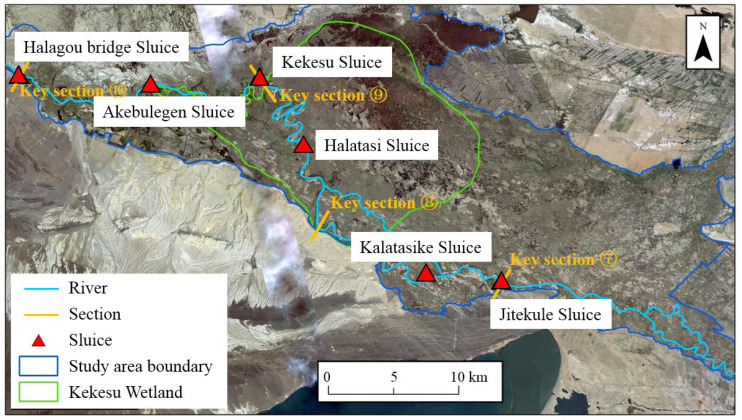
The locations of the sluices in the middle course of the Irtysh River.

According to the calculation results for the water level of the main stream under different flood peak flows (Table 6), the areas that were less likely to be flooded were mainly distributed in the middle course of the Irtysh River. Sluices are effective at alleviating the impacts of insufficient ecological water supply and improving the ecological effects when used for reservoir regulation. They can raise the water level of the channels and make flooding more likely. Based on the distribution of the valley forests and grasslands, sluices could be constructed in the Kekesu Wetland and downstream of the junction of the Irtysh River and the Kelan River. The locations of the sluices are shown in Figure 19.

#### 3.5.2. Optimization of the Ecological Regulation Rules

Sluices can raise the water level of channels, making it easier for flooding to occur on the floodplain and improving the inundation effect in the case of a reduced flood peak duration or flood peak flow. To optimize the reservoir regulation rules in the case of constructing ecological sluices, two objectives should be considered: guaranteeing the flood peak flow and the flood peak duration. When the flood peak flow is guaranteed, the flood duration will be reduced accordingly. When the flood peak duration is guaranteed, the flood peak flow in the 635 Reservoir will be reduced to meet the flood peak duration requirement of 168 h. When the ecological water supply volumes of the 635 Reservoir were 528, 482, 398, and 301 million m^3^, the flood peak durations in the case of guaranteeing the flood peak flow were 141, 128, 105, and 78 h, respectively, while the flood peak flows in the case of guaranteeing the flood peak duration were 880, 800, 655 and 500 m^3^ s^−1^, respectively.

Table 10 shows the effects of sluices on the improvement of the flooded area. As can be seen, the smaller the ecological water supply volume, the higher the effects of the sluices. When the ecological water supply volume is high, considering the flood peak flow as the objective could make the sluices more effective. When the water supply volume was 528 million m^3^, the inundation area increased by 5.5 km^2^; when the water supply volume was 482 million m^3^, the inundation area increased by 4.7 km^2^. Additionally, when the ecological water supply volume is small, considering the flood peak duration as the objective can make the sluices more effective. When the water supply volume was 398 million m^3^, the inundation area increased by 10.3 km^2^; when the water supply volume was 301 million m^3^, the inundation area increased by 37.6 km^2^. Therefore, when sluices are used together with reservoir regulation, rules can be implemented that guarantee the flood peak flow when the ecological water supply volumes are 528 and 482 million m^3^ and that guarantee the flood peak duration when the ecological water supply volumes are 398 and 301 million m^3^.

#### 3.5.3. Improvement Effects of Ecological Regulation

**Figure 20:**
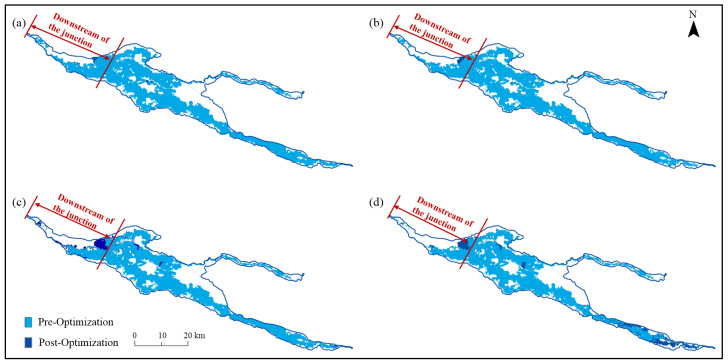
Impacts of the sluices on the maximum inundation range under different ecological water supply volumes: (**a**–**d**) indicate 528, 482, 398 and 301 million m^3^, respectively.

In terms of the inundation range, through a comparison between the floodplain situation before and after the optimization of the ecological rules under different ecological water supply volumes (as shown in Figure 20), it can be observed that the improvement effects of ecological regulation were mainly reflected in the increase in the floodplain range in the valleys on both upstream banks and downstream of the junction of the Irtysh River and the Kelan River. Furthermore, the lower the ecological water supply volume, the more significant the improvement effects. When the water supply volume was reduced to 301 million m^3^, guaranteeing the flood peak duration led to a significant increase in the inundation range of the valleys on both banks upstream of the Irtysh River.

In terms of the inundation duration (as shown in Table 11), when the ecological water supply volumes were 528 and 482 million m^3^, the areas that were flooded for 6 d were the largest and covered an area of 173.0 and 174.2 km^2^, respectively, which increased by 5.0 and 6.7% compared to that before the optimization. Then, when the ecological water supply volumes were 398 and 301 million m^3^, the areas that were flooded for 7 d were the largest and covered areas of 141.8 and 124.7 km^2^, respectively, and the ranges that were flooded for more than 7 d increased by 60.8 and 75.0% compared with that before the optimization.

## 4. Discussion

Water is a non-biological component of ecosystems, and hydrological processes are the main driving force for topography shaping, environmental evolution, and ecosystem succession. At present, a large number of water conservancy projects have been constructed and put into operation, which has had adverse effects on the ecosystem.

In this study, the theory of ecological reservoir regulation coupled with hydrological and ecological processes was studied, using the Irtysh River Basin as a case study. In a broad sense, ecological reservoir regulation aims to meet the ecological needs of the river as much as possible by regulating the discharge of the reservoir. However, it is difficult to completely restore the natural hydrological processes of dammed rivers due to the various social service functions of reservoirs. Therefore, in a narrow sense, ecological reservoir regulation aims to restore the key hydrological processes of river ecosystems according to conservation objectives [47]. The previous studies on ecological regulation mainly focused on the regulation of reservoir water volume, taking ecological base flow as a constraint condition. For example, Xu [48] used the Tennant method to calculate the ecological flow requirements of channels and applied them to the multi-objective operation strategy for reservoirs; Feng et al. [49] used the chaotic initialization method to balance the generation benefit and ecological requirement. However, these studies have not considered the ecological demand processes of conservation objectives.

Ecological restoration should provide hydrological processes similar to key natural runoff as much as possible [50]. The unique feature of this paper is its taking the hydrological process demands as the ecological objectives. The objective of ecological regulation in the Irtysh River Basin is to restore the hydrological processes of flooding to satisfy the water demand for the reproductivity and growth of the valley forests and grasslands. A 2D hydrodynamic model was used to establish the response relationship between the river hydrological and reservoir discharge processes and to determine the key parameters for reservoir regulation. Both the simulation of the 2D hydrodynamic model and the practice of ecological dispatching showed that the research methods and results were reasonable and could effectively restore the ecological environment by saving water resources.

The ecological regulation theory and methods that were examined in this paper could also apply to other basins and their conservation objectives. In the restoration and reconstruction of damaged wetlands, the restoration of hydrological conditions is a major technical measure, and water replenishment technology can eliminate the influence of water shortages and salinity by increasing the water supply [51,52]. In stream restoration projects, water replenishment is the main means of restoration. Thus, the water quality and quantity coupling hydrodynamic model is an effective tool for simulating and analyzing the water quality improvement effects [53], promoting the health of aquatic ecosystems, and restoring various habitats [54]. Additionally, in the management of the water levels of lakes, their control is a more important problem [55]. Toward that end, the hydrodynamic model can effectively determine the appropriate control measures such as water diversion and floodgate control [56,57].

To further optimize ecological reservoir regulation and restoration, the relationship between the water demands of the conservation objectives and water supply volumes should be the focus in subsequent studies. The establishment of a relationship among the ecological water demand, ecological water supply, and ecological effects will be of great significance in improving the theory and implementation of ecological restoration.

## 5. Conclusions

The core method for achieving the protection and remediation of valley forests and grasslands through ecological regulation is to restore the hydrological processes to meet the water demand during the key ecological stages by building artificial flood peaks through reservoir regulation. In this study, with the protection and remediation of valley forests and grasslands in the arid regions of the basin of the Irtysh River as a case study, the problem of the insufficient understanding of the hydrological process demands at the key ecological stages and the difficulty in evaluating the ecological regulation effects were addressed using a hydrodynamic model. Based on the model, the impacts of insufficient ecological water supply on ecological regulation were analyzed, and countermeasures were proposed. The main conclusions included that (1) to meet the demand for the essential hydrological processes of the valley forests and grasslands, the ecological regulation flood peak flow of the 635 Reservoir should be 1000 m^3^ s^−1^, the flood peak duration should be 168 h, and the total regulated water volume should be 605 million m^3^. Ecological regulation can ensure an inundation area of 668.5 km^2^, accounting for 64.3% of the core area of regulation in the Irtysh River Basin. Most of the areas were inundated for 4–8 d, mostly in the valleys on both banks of the middle course and the Kekesu Wetland, with the area that was inundated for 7 d being the largest and covering 159.9 km^2^. Ecological regulation can effectively restore the valley forest and grassland ecosystem by saving water resources, based on the effects of practices from 2016 to 2019. The average vegetation coverage can be restored to 74.3%, and more than 80% of the study area showed an increase in grass yield. Additionally, (2) insufficient ecological water supply will lead to the reduction of the inundation area and duration, significantly affecting the ecological regulation effect. The calculation results that were obtained under four conditions of insufficient ecological water supply, namely, 528, 482, 398, and 301 million m^3^, showed that the inundation area was reduced by 2.8, 5.1, 10.3%, and 19.3%, respectively, and the inundation duration was generally shortened. The area from the 635 Reservoir to Yinejihai Engineering and downstream of the junction of the two rivers showed the most significant changes. Furthermore, (3) the construction of sluices and the optimization of the reservoir regulation rules can effectively alleviate the impact of an insufficient ecological water supply, and the less the ecological water supply, the more significant the improvement effects. When the ecological water supply volumes are 528 or 482 million m^3^, the regulation rules should be implemented with the flood peak flow guaranteed, and when the ecological water supply volumes are 398 or 301 million m^3^, the regulation rules should be implemented with the flood peak duration guaranteed.

This study is of important theoretical significance and reference value for the study of ecological protection and remediation in arid regions, but the response mechanism of the ecological protection objectives to the hydrological processes still needs to be established. Therefore, further improvement through long-term practice and monitoring is required in the future.

## Figures and Tables

**Table 1:** The key sections of the study area.

Section Number	Name	Section Number	Name
1	635 Reservoir	7	Zhonghui Bridge
2	Sarbulak	8	Halatielieke
3	Beitun Bridge	9	The junction of the Irtysh River and Kelan River
4	7 km Section		
5	14 km Section	10	Halagou Mudao Bridge
6	Yinejihai Engineering	11	Burqin Hydrological Station

**Table 2:** Data used in this study.

Data Type	Data Content
Geological data	Channel section, 2.5 m contour line, measured elevation points with a precision of 1 km, DEM data with a resolution of 15 m
Hydrological data	2 h outflow processes of the 635 and Keaijiaer reservoirs, daily water levels and flows of the Burqin Hydrological Station, and daily flows of Yinejihai Engineering
Meteorological data	Daily evaporation and infiltration observation data
Remote sensing image data	Remote sensing imaging from the SPOT-6 satellite and Environment-1 satellite during the corresponding period

Abbreviations: DEM, Digital Elevation Model; SPOT, Systeme Probatoire d’Observation de la Terre.

**Table 3:** The simulation results for the flood peak flow at the monitored sections in 2016.

Section Location	Peak Occurrence Time	Measured Flow (m^3^ s^−1^)	Simulated Flow (m^3^ s^−1^)	Flow Error (%)	*NSE*
Key section ②	6 June 2016	1120	1140	2.1	0.85
Key section ⑩	10 June 2016	811	878	8.3	0.85
Key section ⑪	15 June 2016	822	918	11.7	0.88

Abbreviation: *NSE*, Nash–Sutcliffe efficiency factor.

**Table 4:** The simulation results for the flood peak flow at the monitored sections in 2017.

Section Location	Peak Occurrence Time	Measured Flow (m^3^ s^−1^)	Simulated Flow (m^3^ s^−1^)	Flow Error (%)	*NSE*
Key section ②	27 May 2017	1020	1060	4.2	0.93
Key section ⑩	2 June 2017	821	870	6.0	0.85
Key section ⑪	24 May 2017	538	525	−2.4	0.94
	3 June 2017	868	847	−2.5	

Abbreviation: *NSE*, Nash–Sutcliffe efficiency factor.

**Table 5:** The measured and simulated inundation areas in the core area.

Date	Inundation Area (km^2^)		Area Error (%)
	Measured	Simulated	
9 June 2016	493.2	492.7	−0.1
12 June 2016	545.4	499.4	−8.5
14 June 2016	569.9	497.9	−12.6
22 May 2017	352.7	395.6	12.2
3 June 2017	471.3	464.3	−1.5

**Table 6:** The calculated water levels of the main stream of the Irtysh River at different flood peak outflows from the 635 Reservoir.

Section Number	Bottomland Elevation (m)	Flood Peak 800 m^3^ s^−1^		Flood Peak 1000 m^3^ s^−1^	
		Calculated Highest water Level (m)	Flooding on the Floodplain	Calculated Highest Water Level (m)	Flooding on the Floodplain
Key section ③	507.64	507.14	×	507.33	×
Key section ④	499.28	499.13	×	499.33	√
Key section ⑤	496.50	496.36	√	496.55	√
Key section ⑦	489.30	488.75	×	489.04	×
Key section ⑧	482.40	482.66	√	482.83	√
Key section ⑨	480.38	480.32	×	480.57	√
Key section ⑩	475.77	475.45	×	475.77	√

The symbol “√” indicates flooding on the floodplain, and the symbol “×” indicates no flooding on the floodplain.

**Table 7:** The area statistics of the inundation duration.

Inundation Duration (d)	Area (km^2^)	Inundation Duration (d)	Area (km^2^)
0	352.0	6	112.9
1	6.7	7	159.9
2	12.0	8	111.5
3	22.4	9	29.6
4	35.0	10	124.2
5	72.9		

**Table 8:** The maximum inundation area under different ecological water supply volumes.

Ecological Water Supply Volume (Million m^3^)	Maximum Inundation Area (km^2^)	Amount of Variation (km^2^)	Rate of Variation (%)
605	668.5	/	/
528	649.9	−18.6	−2.8
482	634.7	−33.8	−5.1
398	599.5	−69.0	−10.3
301	539.7	−128.8	−19.3

The symbol “/” indicates inappropriate data.

**Table 9:** The inundation durations and areas under different ecological water supply volumes (unit: km^2^).

Water Supply Volumes (Million m^3^)	Inundation Duration (d)								
	1	2	3	4	5	6	7	8	9
605	6.7	12.0	22.4	35.0	72.9	112.9	**159.9**	111.5	29.6
528	4.7	12.7	22.0	36.4	81.1	**164.8**	140.8	70.2	27.6
482	5.9	11.2	21.6	38.8	117.7	**163.3**	113.9	58.9	27.1
398	7.3	7.9	21.8	81.9	**153.9**	122.1	89.8	39.0	26.9
301	5.0	12.4	74.9	**116.2**	104.5	98.2	75.5	19.2	26.8

The bold numbers indicate the area of inundation duration with the largest proportion.

**Table 10:** The effects of the sluices on the maximum inundation areas.

Ecological Water Supply Volume (Million m^3^)	Inundation Area (km^2^)						
	Without Ecological Sluices	With Sluices					
		Guaranteeing the Flood Peak Flow	Variation in the Area	Rate of Variation (%)	Guaranteeing the Flood Peak Duration	Variation in the Area	Rate of Variation (%)
528	649.9	655.3	**5.4**	**0.8%**	652.9	3.0	0.5%
482	634.7	639.4	**4.7**	**0.7%**	621.2	−13.5	−2.1%
398	599.5	609.0	9.5	1.6%	609.7	**10.2**	**1.7%**
301	539.7	549.9	10.2	1.9%	577.3	**37.6**	**7.0%**

The bold numbers indicate the better effect of the two schemes under different water supplies.

**Table 11:** The inundation durations and areas under different ecological water supply volumes after optimization (unit: km^2^).

Water Supply Volumes (Million m^3^)	Inundation Duration (d)								
	1	2	3	4	5	6	7	8	9
528	4.6	12.7	20.1	32.1	81.5	**173.0**	139.5	78.6	21.2
482	5.7	10.7	20.3	34.0	117.2	**174.2**	113.9	64.6	20.6
398	12.8	16.1	18.8	34.4	89.5	93.4	**141.8**	86.0	22.6
301	15.6	14.5	22.5	55.1	71.0	92.9	**124.7**	66.6	21.3

The bold numbers indicate the area of inundation duration with the largest proportion.

## Data Availability

The initial data for the study and the main results are available from the corresponding author upon request; however, approval will be required from the funding agency.
